# RETRACTION: Therapeutic Efficacy of Platelet‐Rich Fibrin on Surgical Site Wound Healing in Patients Undergoing Oral Carcinoma Resection: A Meta‐Analysis

**DOI:** 10.1111/iwj.70511

**Published:** 2025-04-21

## Abstract

**RETRACTION:** LongT. , LiC. , XuF. , and XiaoJ. , “Therapeutic Efficacy of Platelet‐Rich Fibrin on Surgical Site Wound Healing in Patients Undergoing Oral Carcinoma Resection: A Meta‐Analysis,” International Wound Journal 21, no. 1 (2024): e14386, 10.1111/iwj.14386.37697485 PMC10784624

The above article, published online on 11 September 2023, in Wiley Online Library (http://onlinelibrary.wiley.com/), has been retracted by agreement between the journal Editor in Chief, Professor Keith Harding; and John Wiley & Sons Ltd. Following an investigation by the publisher, all parties have concluded that this article was accepted solely on the basis of a compromised peer review process. The editors have therefore decided to retract the article. The authors did not respond to our notice regarding the retraction.

**RETRACTION:** T. Long , C. Li , F. Xu , and J. Xiao , “Therapeutic Efficacy of Platelet‐Rich Fibrin on Surgical Site Wound Healing in Patients Undergoing Oral Carcinoma Resection: A Meta‐Analysis,” International Wound Journal 21, no. 1 (2024): e14386, 10.1111/iwj.14386.37697485 PMC10784624

The above article, published online on 11 September 2023, in Wiley Online Library (http://onlinelibrary.wiley.com/), has been retracted by agreement between the journal Editor in Chief, Professor Keith Harding; and John Wiley & Sons Ltd. Following an investigation by the publisher, all parties have concluded that this article was accepted solely on the basis of a compromised peer review process. The editors have therefore decided to retract the article. The authors did not respond to our notice regarding the retraction.

